# Foot arch morphology and lower-limb biomechanical characteristics in university students: a cross-sectional multifactorial analysis of 1,078 participants

**DOI:** 10.1038/s41598-026-38118-1

**Published:** 2026-02-05

**Authors:** Zhiyi Xu, Yu Lin, Yiquan Chen, Jie Lu, Yihui Jiang, Jiahao He, Liyuan Yu, Wensheng Miao, Jinhui Yan, Xiangdong Wang

**Affiliations:** 1 School of Physical Education, Jimei University, 107 Yinjiang Road, Xiamen, 361021 Fujian China; 2 China Research Center on Aging, No. 28, Xinniao Kou Wai Street, Xicheng District, Beijing, 100088 China

**Keywords:** Foot arch morphology, Sports injury, Biomechanics, Hallux valgus, Calcaneal angle index, Postural stability, Plantar pressure, Hurst index, Anatomy, Diseases, Health care, Medical research, Risk factors

## Abstract

To examine how medial longitudinal arch morphology relates to a multidomain set of static biomechanical correlates—covering structure (forefoot/rearfoot alignment), loading/pressure behavior, arch “spring-like” function, and postural-control surrogates—as a pragmatic screening framework in university students. In this cross-sectional study, 1,078 university students (569 males, 509 females; mean age 20.18 ± 1.43 years) underwent standardized quiet-standing assessments. Arch height, hallux valgus angle, and heel valgus angle were measured using a 3D foot scanner, and plantar-pressure–derived outcomes (including arch elasticity index, AEI, and pressure recovery rate, PRR, reflecting static elastic deformation and unloading recovery behavior) were obtained using a pressure platform. Force-plate center-of-pressure (COP) time series were used to derive coordination asymmetry index (CAI; COP-based inter-limb asymmetry) and the Hurst exponent (temporal structure/long-range correlation of COP fluctuations). Left and right feet were analyzed separately for laterality-specific associations. Linear regression models evaluated continuous (arch height) and categorical (arch type; reference = normal) predictors, with sex included as a covariate; bilateral arch-height difference was modeled as a predictor of stability-related outcomes (center-of-mass displacement and mediolateral COP deviation). False discovery rate control was applied within outcome families. Arch morphology showed consistent associations with static alignment and function. Lower-arched profiles were associated with larger hallux valgus and greater heel valgus (eversion) angles, alongside lower AEI and PRR, indicating a less favorable static “spring” and unloading-recovery profile. A subset of high-arched feet also demonstrated less favorable forefoot alignment patterns, suggesting that both excessively low and excessively rigid arches may relate to forefoot mechanics, although estimates for less prevalent categories should be interpreted cautiously. Greater bilateral arch-height asymmetry was associated with larger stability-related displacements and COP deviations, and arch abnormalities/asymmetry corresponded to altered COP-derived coordination signatures (higher CAI and shifts in Hurst exponent), indicating differences in postural-control behavior under quiet standing. In university students, arch morphology and bilateral asymmetry are associated with multiple domains of static foot biomechanics, spanning alignment, pressure/elastic-function indices, and COP-derived postural-control surrogates. These findings support considering a combined screening perspective of arch type + function (AEI/PRR) + asymmetry, while emphasizing that longitudinal and interventional studies are required to determine clinical utility, actionable thresholds, and relevance to symptoms or injury outcomes.

**Supplementary Information:**

The online version contains supplementary material available at 10.1038/s41598-026-38118-1.

## Introduction

The morphology of the foot arch (hereafter “arch,” referring to the medial longitudinal arch) is a fundamental structural feature that modulates load transfer, shock attenuation, and energy storage in the lower limb. Variations in arch type—commonly categorized as planus (flatfoot), neutral, and cavus (high arch)—have been associated with distinct plantar loading patterns and lower-limb biomechanics, and are frequently discussed in relation to injury-relevant mechanical stress and functional complaints^1–3^.

Biomechanical studies have shown that planus and cavus feet present different rearfoot kinematics and loading strategies during gait. For example, compared with neutrally aligned feet, planus feet demonstrate greater hindfoot eversion during stance, whereas cavus feet typically exhibit a more inverted rearfoot pattern^2^. Notably, the reported sign (positive/negative) of inversion–eversion angles can vary across coordinate systems; therefore, the key interpretation is the relative magnitude and timing of eversion within stance, with planus feet often reaching peak eversion around mid-stance and tending to do so earlier within the stance phase^2^.

Beyond kinematic differences, several arch-related structural and loading features have been linked—primarily in associative evidence—to specific overuse conditions. Increased navicular drop has been reported as being associated with a higher occurrence of medial tibial stress syndrome, suggesting that altered medial loading and shock dissipation may be involved^4^. In addition, posterior tibial tendon dysfunction can shift the center of pressure posteriorly and increase medial forefoot loading, potentially contributing to progressive arch collapse and secondary deformity^5^. While these findings do not establish causation, they support the rationale that arch morphology is a clinically relevant descriptor of foot structure and loading behavior^4,5^.

The biomechanical relevance of arch posture may extend along the kinetic chain. In static alignment, excessive pronation has been associated with increases in tibial and hip internal rotation and anterior pelvic tilt^6^. During gait, musculoskeletal simulation studies further suggest that hyperpronation can alter the timing and intensity of lumbopelvic muscle forces and increase anterior pelvic tilt across the stance phase, providing a plausible mechanical pathway linking foot posture to proximal function^7,8^. Consistently, population-based evidence has reported associations between foot posture/function and low back pain, with a stronger relationship observed in females^3^.

Arch morphology and balance performance can also be influenced by individual characteristics such as body mass, underscoring the multifactorial nature of foot biomechanics in young adults^9^.

University students constitute a practical target population for foot-structure screening because they commonly participate in recreational sports and structured physical education. Epidemiological data indicate that physical activity–related injuries are not rare in this group; for example, a 1-year prospective study in Chinese college students reported that 31.0% experienced at least one physical activity–related injury during follow-up^10^. Meanwhile, existing student-focused studies have often examined plantar pressure descriptors or compared selected gait features between flatfoot and normal-arch groups^11,12^, and trials have explored exercise or insole interventions for flexible flatfoot^13^. However, large-sample analyses that simultaneously characterize arch-type distribution and integrate multiple injury-relevant biomechanical domains—such as bilateral symmetry, postural stability, and elastic energy-related indices—remain limited^3,4,14,15^.

To address these gaps, the present cross-sectional study analyzed arch screening data from 1,078 university students using a multifactorial statistical framework. We aimed to quantify the associations between arch morphology (including bilateral symmetry) and a set of lower-limb biomechanical characteristics measured under standardized static conditions, focusing on plantar loading, postural stability–related parameters, and elastic function indicators. Importantly, our objective is to describe biomechanical correlates of arch structure rather than to infer causality or to predict injury occurrence.

## Methods

### Study design​​

This cross-sectional study investigated associations between foot arch morphology and a set of static lower-limb biomechanical characteristics in university students. All assessments were conducted in the Sports Biomechanics Laboratory of Jimei University (Xiamen, China) between October and December 2024. The study was approved by the Ethics Committee of the School of Physical Education, Jimei University, and all participants provided written informed consent prior to testing.

### Participants and recruitment​​

Participants were recruited during the university’s annual student physical fitness and health assessment program, which includes undergraduate students from multiple schools/colleges within the university and represents students from diverse regions across China. Students were invited to participate on a voluntary basis and were enrolled if they met the eligibility criteria.

**Fig. 1:**
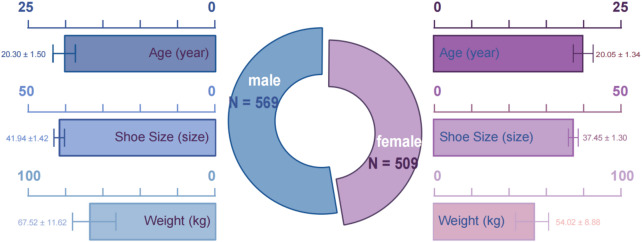
Participant Demographic Characteristics​.

Inclusion criteria were: (1) no history of lower-limb fracture or surgery; (2) no serious foot or lower-limb injury in the past six months; and (3) ability to complete the required static assessments. Exclusion criteria were: (1) known neurological conditions affecting balance or foot posture assessment; and (2) inability to complete the testing procedures. Sex, age, body weight, and shoe size were recorded to characterize the sample and are presented in Fig. 1.

### Data collection and measurements​

#### 3D foot morphology (arch height and alignment)

Foot arch morphology, hallux valgus angle, and heel valgus angle were measured using the iFEET Neo 3D foot scanner (iSUN3D, China) under bipedal weight-bearing static conditions. The device captures a three-dimensional foot model using structured-light scanning (point cloud density ≈ 28/cm²). Participants stood barefoot in an anatomical neutral posture on the glass platform for 10 s, with both feet fully within the scanning area (400 mm × 400 mm × 150 mm). Two trained assessors independently performed measurements.

Arch type thresholds were defined according to the device report output (manufacturer-defined): high arch (X > 9 mm), normal arch (5 < X ≤ 9 mm), flatfoot (3.5 < X ≤ 5 mm), flatfoot+ (2 < X ≤ 3.5 mm), and flatfoot++ (X ≤ 2 mm). Hallux valgus categories were defined as: standard (X ≤ 15°), valgus (15° < X ≤ 30°), valgus+ (30° < X ≤ 40°), and valgus++ (X > 40°). Heel valgus angle categories were defined as: severe varus (X ≤ − 8°), varus (− 8° < X ≤ − 4°), normal (− 4° < X ≤ 4°), valgus (4° < X ≤ 8°), and severe valgus (X > 8°). The scanner-based measurement approach in weight-bearing conditions is consistent with prior validation work showing millimeter-level repeatability of 3D foot scanning under load^16,17^.

### Plantar pressure analysis​​

Plantar pressure distribution was assessed using the iGAIT MAX 3D pressure platform (iSUN3D, China; 400 Hz sampling frequency). The system integrates piezoresistive sensors (four gold-plated sensors/cm²; 2.5 DPI resolution; maximum pressure of 150 N/cm²) to quantify center-of-mass displacement, mediolateral pressure center deviation, arch elasticity index (AEI), and pressure recovery rate (PRR). Participants completed three 10-second static standing trials on the pressure plate while maintaining natural posture. Data from repeated trials were averaged to minimize intra-subject variability and to obtain a stable point estimate under standardized quiet-standing conditions. Trial-level outputs were not retained in the curated analytical dataset; therefore, within-session reliability metrics (e.g., ICC/SEM) are not reported in the present study.

### Postural stability assessment​​

Coordination Asymmetry Index (CAI) and Hurst exponent were derived from force plate data (Kistler 9286B, Switzerland; 100 Hz sampling frequency). Participants stood barefoot at the force plate center for 15 s during data acquisition.

### Biomechanical parameter calculations​

***CAI.*** The $\:\mathrm{CAI}$ evaluates bilateral symmetry of the center of pressure (COP) during static standing, calculated as:

1 $$CAI = \frac{{\left| {CO{P_L}0COP{}_R} \right|}}{{\left( {CO{P_L} + CO{P_R}} \right)/2}} \times 100\%$$

where $\:{\mathrm{COP}}_{\mathrm{L}}$ and $\:{\mathrm{COP}}_{\mathrm{R}}$ denote left and right foot pressure center coordinates derived from spatial centroids or moment-based calculations. Elevated $\:\mathrm{CAI}$ values indicate elevated weight-bearing asymmetry and compromised postural stability.

***Hurst Exponent.*** The Hurst exponent ($\:\mathrm{H}$) characterizes long-range correlations in COP time-series data through Rescaled Range ($\:\mathrm{R}\mathrm{/}\mathrm{S}$) analysis.

2 $${\text{R/S(n) = }}\frac{{{\mathrm{R(n)}}}}{{{\mathrm{S(n)}}}}\alpha {{\mathrm{n}}^{\mathrm{H}}}$$

where $\:\mathrm{n}$ represents time-series length, $\:\mathrm{R}\mathrm{(}\mathrm{n}\mathrm{)}$ the range of cumulative deviations, and $\:\mathrm{S}\mathrm{(}\mathrm{n}\mathrm{)}$ the standard deviation (SD). The exponent $\:\mathrm{H}$ is obtained as the slope of the linear regression between $\:\mathrm{log(}\mathrm{R}\mathrm{/}\mathrm{S}\mathrm{)}$ and $\:\mathrm{log(}\mathrm{n}\mathrm{)}$. An $\:\mathrm{H}$ value of 0.5 indicates uncorrelated random walk dynamics, $\:\mathrm{H}$ > 0.5 indicates persistent trends, and $\:\mathrm{H}$ < 0.5 reflects anti-persistent behavior. Analysis utilized a stabilized 10-second window from the mid-portion of vertical pressure data to exclude transient postural adjustments.

### Environmental controls​​

All tests were conducted in a climate-controlled laboratory (22–24 °C, 40%–60% humidity) to standardize environmental influences on biomechanical measurements.

### Statistical analysis​​

Analyses were performed in SPSS (version 29.0; IBM Corp., USA). Continuous variables are summarized as mean ± SD and categorical variables as counts and percentages.

To evaluate laterality-specific associations, left and right foot outcomes were analyzed separately. For continuous predictors, simple linear regression assessed relationships between unilateral arch height (mm) and ipsilateral hallux valgus angle (°) or heel valgus angle (°). For bilateral asymmetry, linear regression assessed bilateral arch height difference (mm) as predictors of center-of-mass displacement (sagittal plane) and mediolateral COP deviation (coronal plane). For categorical predictors, arch type was entered as dummy-coded variables (reference = normal arch), and multivariable linear regression was used to estimate adjusted associations with hallux valgus, heel valgus angle, AEI, and PRR, with sex included as a covariate.

All predictors were entered simultaneously in each model (the “Enter method” in SPSS; i.e., no stepwise selection). Multicollinearity was evaluated using variance inflation factors (VIF).

Statistical significance was evaluated using two-tailed tests with α = 0.05. When multiple related outcomes were tested, false discovery rate (Benjamini–Hochberg) control was applied within each outcome family.

## Results

### Morphological characteristics of the arch

**Fig. 2:**
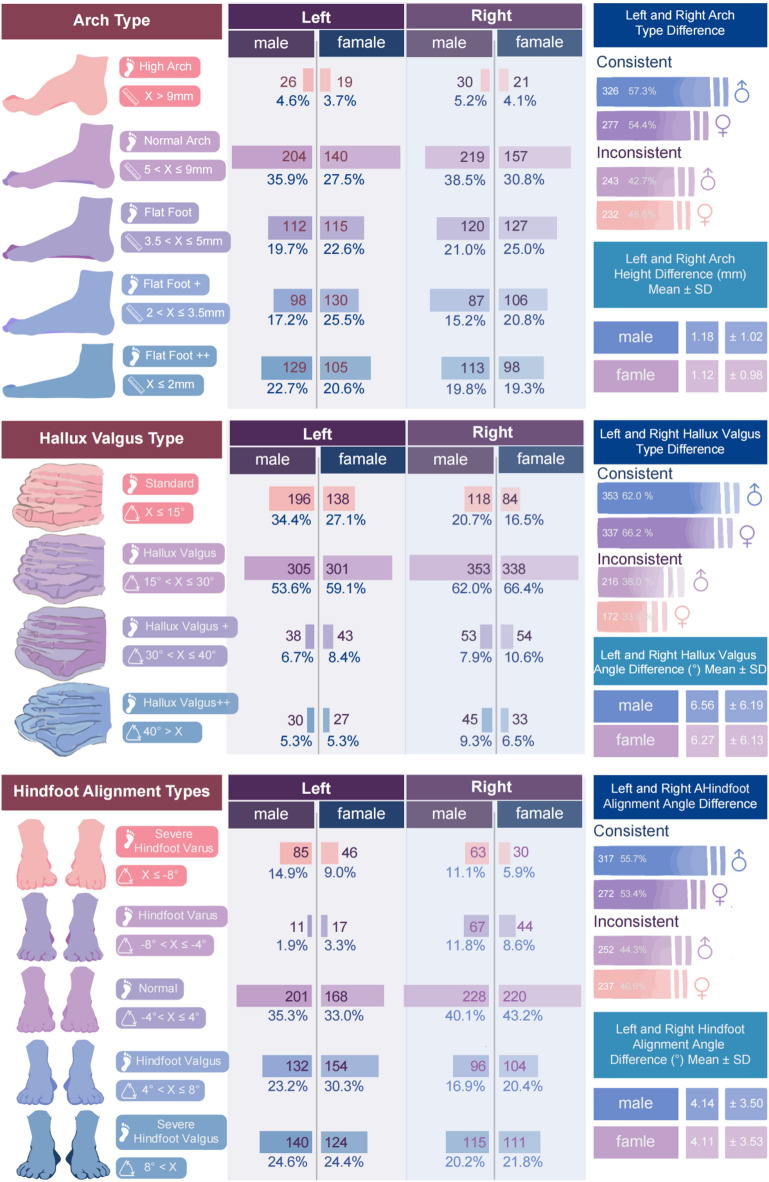
Arch Morphological Characteristics of Participants.

To contextualize the subsequent regression coefficients, Table 1 provides the descriptive statistics (mean ± SD) of key foot morphology and arch-function measures by sex and side. Arch-type distributions and bilateral concordance/asymmetry indices are summarized in Fig. 2.

Descriptive statistics were used to analyze arch height, hallux valgus angle, and heel valgus angle by laterality and sex (Fig. 2). The distribution of left arch types was as follows: high arch 4.2% (male, 4.6%; female: 3.7%), normal arch 31.9% (male, 35.9%; female, 27.5%), flatfoot 21.1% (male, 19.7%; female, 22.6%), flatfoot + 21.2% (male, 17.2%; female, 25.5%), and flatfoot + + 21.7% (male, 22.7%; female, 20.6%). For the right foot, the proportions were as follows: high arch, 4.7%; normal arch, 34.9%; flatfoot, 22.9%; flatfoot+, 17.9%; and flatfoot++, 19.6%. Regarding hallux valgus, 56.2% of left feet and 64.1% of right feet exhibited valgus deformity (15° < X ≤ 30°). A higher prevalence was observed in females for the right foot (66.4% vs. 62.0% in males). For the heel valgus, 34.2% of left feet and 41.6% of right feet were classified as standard, and females showed a higher incidence of left heel valgus (30.3% vs. 23.2% in males). The consistency rate of arch types between left and right feet was 55.9% (male, 57.3%; female, 54.4%), and the average arch height difference was 1.15 ± 1.00 mm (male, 1.18 ± 1.02 mm; female, 1.12 ± 0.98 mm). The consistency rate for hallux valgus was 64.0%, and the average angle difference was 6.38 ± 6.16° (male, 6.56° ± 6.19°; female, 6.17° ± 6.13°). The consistency rate for heel valgus was 54.6%, with an average angle difference of 4.12° ± 3.51° (male, 4.14° ± 3.50°; female, 4.11° ± 3.53°).

**Table 1:** Descriptive characteristics and static Biomechanical measures by sex and side (mean ± SD). Male *n* = 569; female *n* = 509; total *n* = 1078.

Variable	Sex	Left (Mean ± SD)	Right (Mean ± SD)	Overall (Mean ± SD)
Foot morphology				
Foot length (mm)	Male	256.40 ± 11.76	255.27 ± 14.22	255.83 ± 13.05
	Female	234.19 ± 10.48	233.49 ± 10.43	233.84 ± 10.46
	Total	245.91 ± 15.74	244.98 ± 16.62	245.44 ± 16.19
Foot width (mm)	Male	97.77 ± 5.04	97.94 ± 5.06	97.85 ± 5.05
	Female	88.65 ± 4.61	89.15 ± 5.04	88.90 ± 4.83
	Total	93.47 ± 6.65	93.79 ± 6.69	93.63 ± 6.67
Dorsum height at tarsal point (mm)	Male	69.32 ± 5.62	68.72 ± 5.31	69.02 ± 5.47
	Female	63.16 ± 7.77	63.41 ± 26.93	63.28 ± 19.81
	Total	66.41 ± 7.39	66.21 ± 19.08	66.31 ± 14.47
Ankle girth (mm)	Male	258.01 ± 35.43	255.64 ± 27.11	256.82 ± 31.55
	Female	248.22 ± 59.76	244.20 ± 56.39	246.21 ± 58.11
	Total	253.39 ± 48.69	250.24 ± 43.82	251.81 ± 46.34
Arch height (mm)	Male	4.41 ± 2.78	4.66 ± 2.75	4.54 ± 2.77
	Female	4.12 ± 2.62	4.30 ± 2.53	4.21 ± 2.58
	Total	4.27 ± 2.70	4.49 ± 2.65	4.38 ± 2.68
Hallux valgus angle (°)	Male	20.16 ± 10.56	24.43 ± 8.66	22.30 ± 9.89
	Female	21.12 ± 10.06	24.54 ± 8.33	22.83 ± 9.39
	Total	20.61 ± 10.33	24.48 ± 8.50	22.55 ± 9.65
Hindfoot alignment angle (°)	Male	3.03 ± 7.62	1.23 ± 6.83	2.13 ± 7.29
	Female	4.05 ± 6.82	2.35 ± 6.25	3.20 ± 6.59
	Total	3.51 ± 7.27	1.76 ± 6.58	2.63 ± 6.99
Plantar pressure				
Foot contact area (mm²)	Male	13807.12 ± 1195.03	13766.35 ± 1272.87	13786.74 ± 1234.19
	Female	11438.03 ± 973.67	11467.59 ± 1022.07	11452.81 ± 997.78
	Total	12688.51 ± 1612.59	12680.94 ± 1632.61	12684.73 ± 1622.26
Peak pressure (kPa)	Male	21.92 ± 2.47	21.91 ± 2.57	21.91 ± 2.52
	Female	20.02 ± 2.61	20.00 ± 2.67	20.01 ± 2.64
	Total	21.02 ± 2.70	21.01 ± 2.79	21.02 ± 2.74
Mean pressure (kPa)	Male	13.71 ± 2.21	13.71 ± 2.23	13.71 ± 2.22
	Female	12.54 ± 2.04	12.60 ± 2.06	12.57 ± 2.05
	Total	13.16 ± 2.21	13.18 ± 2.22	13.17 ± 2.21
Arch function				
Arch elasticity index (AEI)	Male	56.46 ± 25.45	58.03 ± 24.56	57.25 ± 25.01
	Female	54.47 ± 23.93	56.11 ± 23.86	55.29 ± 23.90
	Total	55.52 ± 24.75	57.12 ± 24.24	56.32 ± 24.50
Pressure recovery rate (PRR)	Male	0.687 ± 0.199	0.707 ± 0.200	0.697 ± 0.200
	Female	0.674 ± 0.186	0.690 ± 0.190	0.682 ± 0.188
	Total	0.681 ± 0.193	0.699 ± 0.195	0.690 ± 0.194
Symmetry and stability				
Pressure-center deviation angle (°)	Male	—	—	4.953 ± 2.032
	Female	—	—	4.986 ± 1.951
	Total	—	—	4.969 ± 1.994
Center-of-mass displacement (mm²)	Male	—	—	21.750 ± 15.771
	Female	—	—	20.187 ± 14.836
	Total	—	—	21.012 ± 15.350
Mediolateral COP deviation (mm)	Male	—	—	−0.554 ± 3.506
	Female	—	—	−0.248 ± 3.593
	Total	—	—	−0.409 ± 3.549
Coordination Asymmetry Index (CAI)	Male	—	—	1.254 ± 0.350
	Female	—	—	1.276 ± 0.333
	Total	—	—	1.264 ± 0.342
Hurst exponent	Male	—	—	0.546 ± 0.192
	Female	—	—	0.522 ± 0.181
	Total	—	—	0.535 ± 0.187

**Fig. 3:**
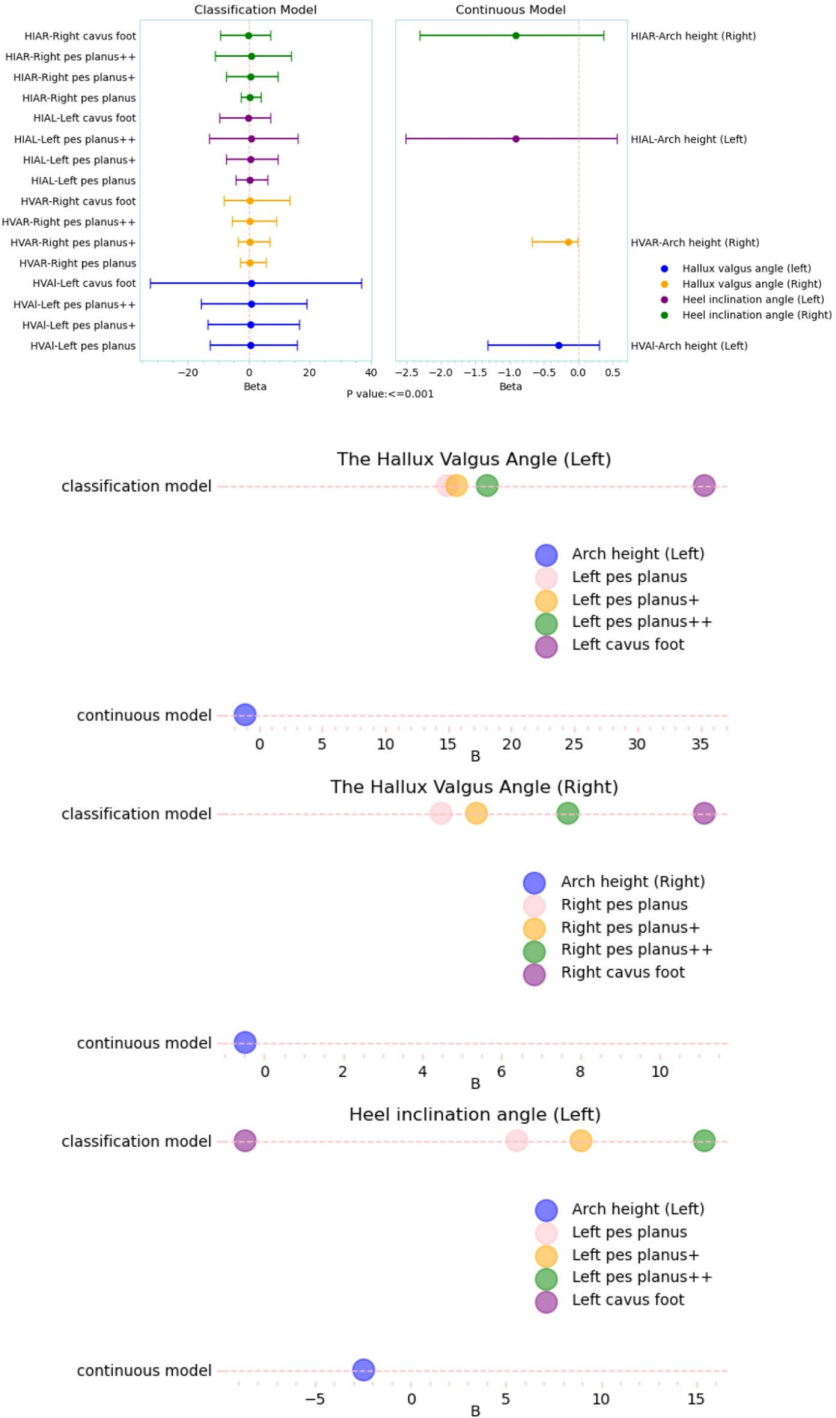

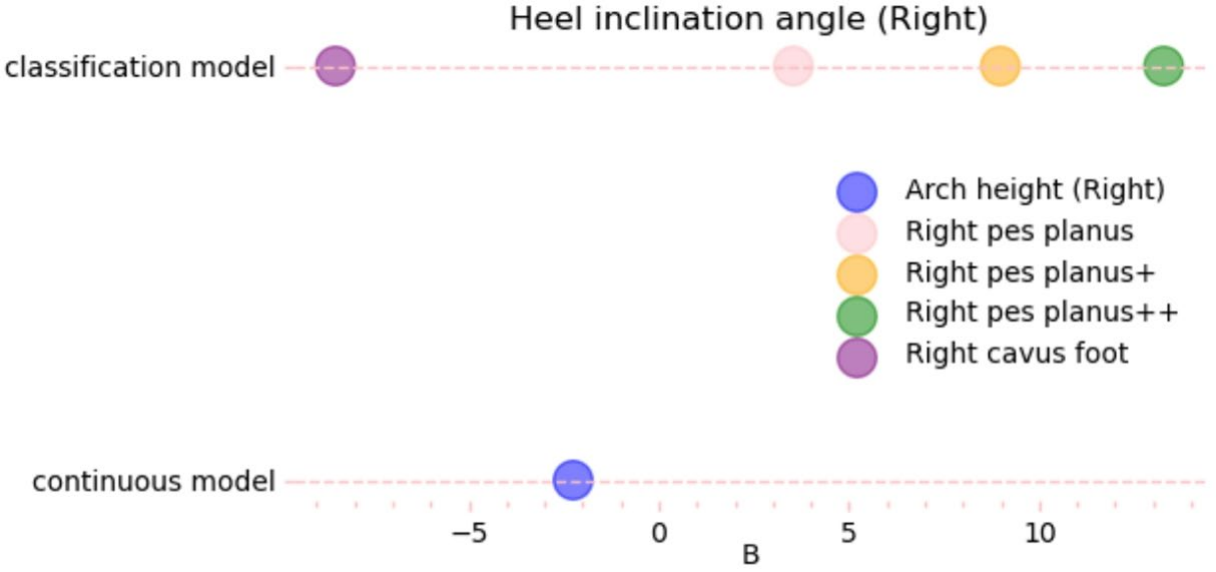
Regression effects of arch morphology on bilateral hallux valgus angle and heel valgus angle. (**A**) Forest plots for the classification model (reference = normal arch) and the continuous model (arch height, mm); dots indicate unstandardized B and lines indicate 95% CI; (**B-E**) B estimates for left/right hallux valgus angle and left/right heel valgus angle (continuous vs. classification). All models were adjusted for sex.

### Heel valgus angle

Changes in arch structure elicited bidirectional regulation of heel 3D biomechanics (Supplementary Table 1). In the left foot continuous model, arch height showed a strong negative correlation with heel valgus angle (*B* = − 2.447, *P* < 0.001), accounting for 82.8% of the variance, which indicated that a 1 mm decrease in arch height led to a 2.45° increase in heel valgus. The categorical model demonstrated a graded response in biomechanical effects due to morphological deformation: flatfoot (*B* = 5.582), flatfoot+ (*B* = 8.943), and flatfoot++ (*B* = 15.412) exhibited a 2.76-fold difference in effect size (*P* < 0.001), whereas high arch showed a correction of 8.66° inversion (*B* = − 8.659, *P* < 0.001). The analysis of the right foot revealed similar trends, but showed a 12.3% reduction in effect size. The effect for flatfoot++ (*B* = 13.272) surpassed the left foot’s normal arch baseline. Additionally, the SD value of the change in heel valgus angle for the left foot (3.01°) was significantly higher than that for the right foot (2.76°), and the largest laterality difference was observed in flatfoot++ (Δ*B* = 2.14°, 95% CI [1.89, 2.39]; Fig. 3).

### Arch height difference and stability

**Fig. 4:**
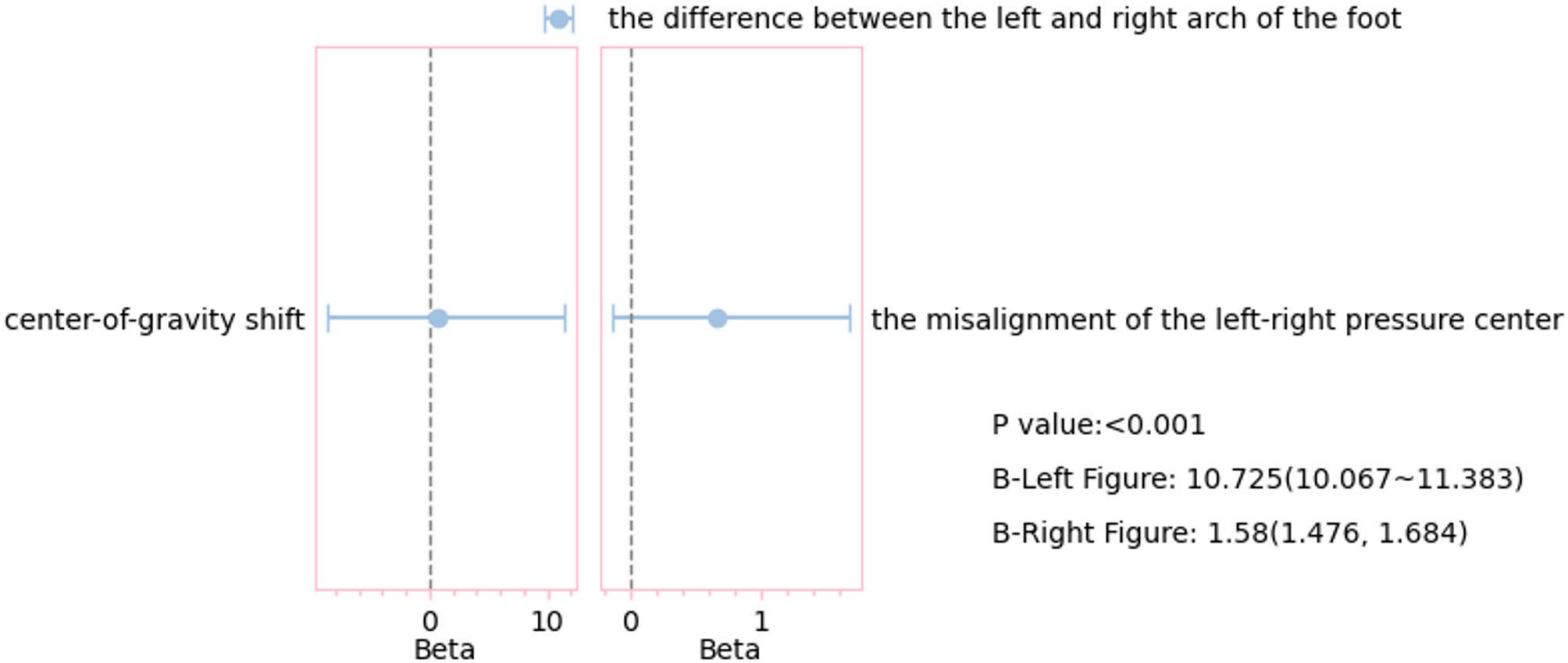
Regression effects of bilateral arch height difference on static stability outcomes​.

Asymmetry in bilateral arches considerably affected postural stability, exhibiting a dose-response relationship (Supplementary Table 2). Regression analysis showed that for every 1 mm increase in arch height difference, center of mass displacement increased by 10.725 mm (95% CI [10.067, 11.383]) and the left–right pressure center misalignment increased by 1.58 mm (95% CI [1.476, 1.684]), with a strength ratio of 6.8:1 (*β* = 0.698 vs. 0.672). These findings suggests that sagittal plane stability is easily influenced by arch asymmetry. Bivariate visualization further revealed that when arch height difference exceeded 3.2 mm, 95% of individuals experienced pressure misalignment exceeding the 5 mm clinical risk threshold. Arch height differences of ≥ 5 mm elicited a 18.6% nonlinear increase in center of mass displacement. The difference in the width of the CI between the two models (1.32 mm vs. 0.21 mm) reflected strong compensatory interference in the sagittal plane, providing dual criteria for personalized orthotics based on spatial sensitivity and mechanical determinism (Fig. 4).

### Arch elasticity index (AEI)

**Fig. 5:**
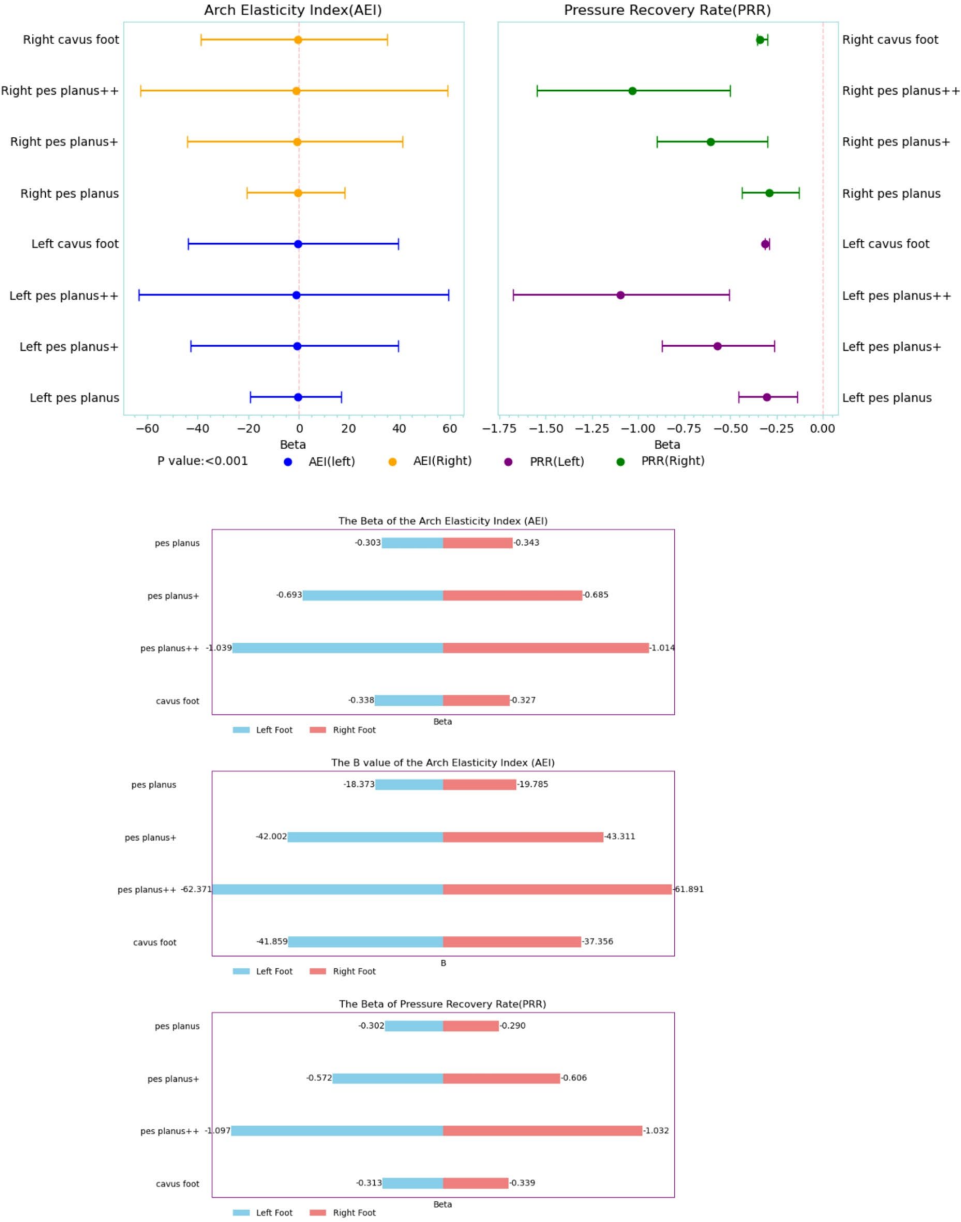

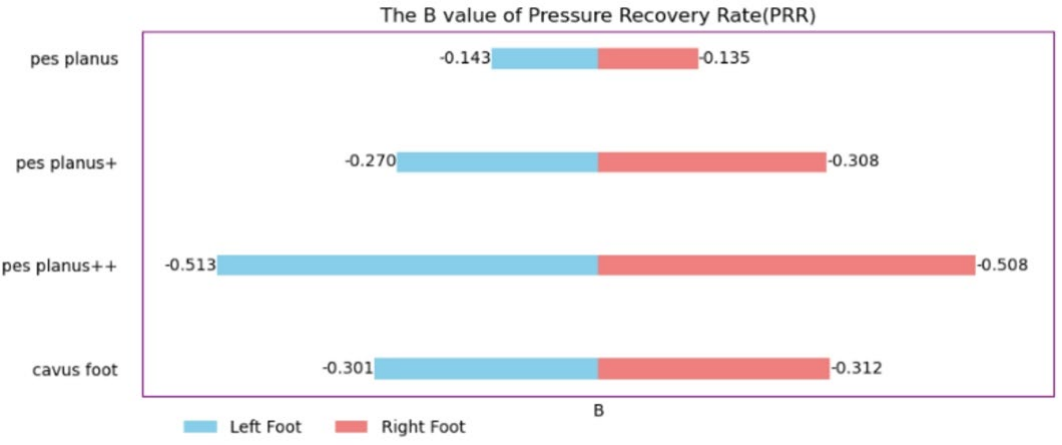
Regression effects of arch morphology on AEI and PRR. (A) Forest plots for the classification model (reference = normal arch) for AEI and PRR; dots indicate unstandardized B and lines indicate 95% CI; (B-E) B and standardized Beta estimates for left/right AEI and left/right PRR.​.

Arch morphological deformities led to a systematic reduction in energy storage function (Supplementary Table 3). In the left foot categorical model, flatfoot + + reduced AEI by 62.371 (95% CI [− 63.379, − 61.363]), which corresponded to 38.7% of the energy storage function of a normal arch (*P* < 0.001). Right foot analysis showed a similar trend, but the high arch effect was diminished by 9.8% (*B* = − 37.356 vs. −41.859). The pyramid chart revealed that left foot deformation caused considerable elastic damage. The laterality difference for flatfoot + + reached 0.48 SD values (Cohen’s *d* = 0.48), and 95% CI (− 0.52, − 0.44) entirely deviated from zero (Fig. 5). The effect size gradient across all deformity types followed a distinct pattern. Flatfoot++ (*d* = 2.01) demonstrated the greatest biomechanical impact, followed by flatfoot+ (*d* = 1.35), high arch (d = 0.98), and flatfoot (d = 0.62).

### Pressure recovery rate (PRR)​​

Arch structural integrity is crucial for shock absorption (Supplementary Table 3). In the left foot, flatfoot + + reduced PRR by 0.513 (95% CI [− 0.518, − 0.507]), indicating that only 51.3% of the normal arch function remained (*P* < 0.001). Right foot analysis showed that flatfoot+ (*B* = − 0.308) and high arch (*B* = − 0.312) had comparable effect sizes, suggesting that lateral arch damage led to equivalent functional loss. The absolute effect size for left foot PRR damage (mean Δ = 0.307) was considerably higher than that for the right foot (Δ = 0.291; Fig. 5). Temporal analysis revealed that the half-life of the left foot pressure recovery curve increased by 23.6% (*P* < 0.001).

### Coordination asymmetry index (CAI)​​

**Fig. 6:**
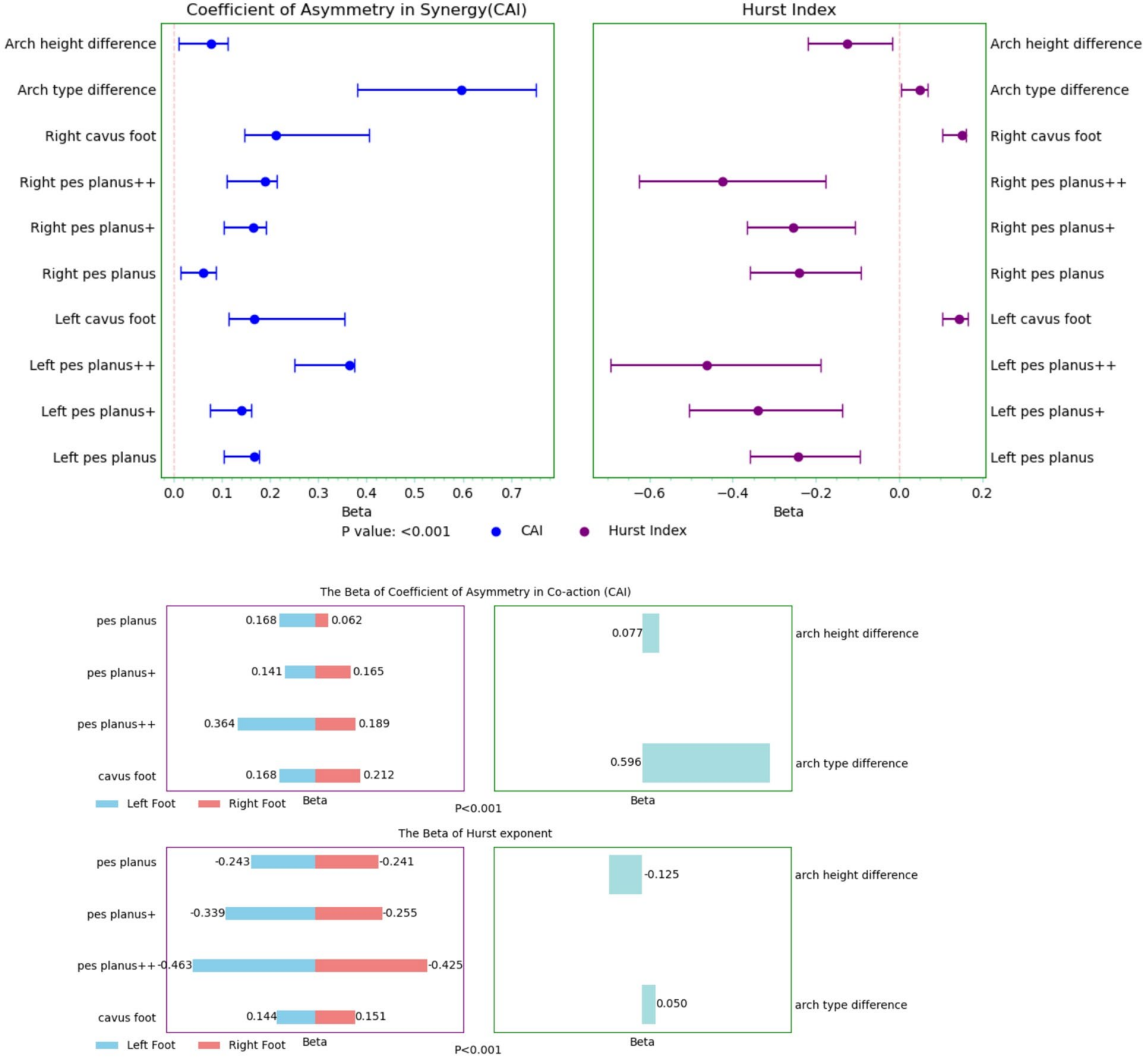
Regression effects of arch morphology on CAI and Hurst index.

Arch abnormalities induced compensatory imbalance in bilateral pressure distribution (Supplementary Table 4). Left foot flatfoot + + increased CAI by 0.302 (*P* < 0.001), equivalent to 3.2 SD of normal arch pressure asymmetry. Right foot high arch had the strongest effect (*B* = 0.341, *P* < 0.001), with its 95% CI (0.277, 0.406) fully covering the left foot flatfoot + + range [0.251, 0.353]. The effect size of bilateral arch type differences (*B* = 0.41, *P* < 0.001) was 15.8 times that of the height difference (*B* = 0.026), accounting for 59.6% of the model’s explanatory power. Additionally, the asymmetry effect for left foot deformities (mean *B* = 0.212) was significantly higher than for the right foot (*B* = 0.170), and the effect strength of type differences increased logarithmically with the degree of deformity (*R*² = 0.872; Fig. 6).

(**A**) Forest plots from multivariable models for CAI and Hurst index; dots indicate unstandardized B and lines indicate 95% CI. (**B–C**) Standardized Beta estimates for CAI and Hurst index, including the effects of arch height difference and arch type disparity. All models were adjusted for sex.​.

### Hurst exponent​​

Changes in arch structure remodeled the long-term stability of pressure distribution (Supplementary Table 4). Left foot flatfoot + + decreased the Hurst index by 0.21 (95% CI [− 0.233, − 0.188]), indicating a 37.2% reduction in long-term correlation of pressure time series (*P* < 0.001). In contrast, right foot high arch produced a positive modulation of 0.133 (*P* < 0.001), with the effect direction being mirror-symmetric to that of the left foot (*r* = − 0.786, *P* = 0.002). The bilateral arch height difference was negatively correlated with the Hurst index (*B* = − 0.023, *P* < 0.001), attenuating 38.7% of the positive effects of type differences. Furthermore, the stability damage for left foot deformities was pronounced. The laterality difference (ΔB) for flatfoot + + was 0.09 (95% CI [0.07, 0.11]), and the effect size gradient following a power law distribution (*R*² = 0.943; Fig. 6).

## Discussion​

### Summary of key findings

In this cross-sectional cohort of university students, foot arch morphology was consistently associated with (i) forefoot and rearfoot alignment characteristics, (ii) static functional indices reflecting arch elasticity and pressure recovery, and (iii) postural/stability surrogates related to bilateral asymmetry. Notably, both low-arched and high-arched feet tended to show less favorable biomechanical profiles in different domains, supporting a “non-linear” (rather than flatfoot-only) view of arch-related screening. These observations should be interpreted as associations under standardized quiet-standing conditions, but they provide mechanistic hypotheses and practical cues for targeted screening and stratified intervention.

### Mechanisms of arch variations in forefoot bony deformities

Our data suggest that variations in arch structure are associated with hallux valgus alignment, with a clearer pattern in the left foot. In general, lower arch profiles were accompanied by a larger hallux valgus angle, which is mechanistically consistent with the classic concept that collapse of the medial longitudinal arch facilitates first-ray instability and first metatarsal pronation/rotation, thereby promoting progressive medial deviation of the first ray and lateral deviation of the hallux^18,19^. Notably, contemporary 3D perspectives emphasize that hallux valgus is a multiplanar deformity, and metatarsal pronation is increasingly considered a key component that may be underestimated by purely planar descriptors^20^.

A second observation was that high-arched feet al.so demonstrated unfavorable hallux valgus alignment in our cohort, supporting a “U-shaped” pattern in which both excessively low and excessively rigid/high arches may be associated with forefoot deformity, albeit via different pathways. For cavus/high-arched feet, a plausible explanation is reduced midfoot compliance and altered load transfer that concentrates plantar pressure toward the forefoot (often around the first and second metatarsal heads), increasing susceptibility to medial forefoot overload and structural deviation, especially in the presence of footwear-related constraints^2,4,19^. Notably, the hallux valgus effect estimate for the high-arch subgroup was unusually large relative to typical clinical ranges and may therefore be unstable, given the small size of this subgroup and the potential influence of extreme or high-leverage observations. Accordingly, this pattern should be interpreted conservatively and verified in independent cohorts (ideally with prespecified influence diagnostics and dynamic measurements such as in-shoe pressure or gait analysis).

Finally, the weaker right-foot associations may reflect laterality-related factors. We did not directly measure foot dominance, but in university populations it is plausible that habitual loading asymmetry and neuromuscular control differences between sides contribute to side-specific buffering or compensation^21^. Accordingly, dominance-related explanations for the stronger left-sided associations remain hypothetical and should be interpreted cautiously. In addition, even under standardized scanning posture, subtle differences in how participants distribute weight across limbs could magnify apparent side differences. Future work that records dominance, habitual sport exposure, and dynamic loading characteristics would help disentangle anatomical from behavioral contributors to laterality.

### Mechanisms of arch variations on rearfoot Tilt

Arch height was also associated with heel valgus angle in a manner that is biomechanically plausible. In general, arch lowering aligns with greater hindfoot valgus/eversion, consistent with coupled motion at the subtalar and midtarsal joints and reduced medial support from the posterior tibial–spring ligament complex^18,22^. This interpretation is compatible with more recent concepts that describe progressive collapse as a 3D alignment problem involving peritalar subluxation and multi-joint malalignment, which can be characterized using weightbearing CT and multi-bone modeling approaches^23^. Such contemporary imaging work supports the view that hindfoot valgus is not an isolated rearfoot phenomenon but part of a broader, integrated deformity pattern linking arch collapse, talar alignment, and midfoot mechanics.

Conversely, high-arched feet showed an opposing tendency toward hindfoot inversion/varus alignment in our dataset. A reasonable mechanistic explanation is that increased longitudinal arch stiffness and changes in plantar soft-tissue geometry (including plantar fascia orientation) can shift load laterally, increasing stress along the lateral column and biasing the rearfoot toward inversion to maintain whole-foot balance^24^. This “opposite-direction” response is consistent with the broader notion that cavus alignment trades off shock attenuation for structural rigidity, potentially altering rearfoot position under load^2^. Importantly, because our measurements were obtained during quiet standing, these associations reflect static alignment correlates; future studies should confirm whether similar coupling patterns are observed during gait (mid-stance and push-off) using synchronized motion capture, in-shoe pressure, and force-platform kinetics.

### Impact of arch asymmetry on postural control and neurological regulation

This study suggests that bilateral asymmetry in arch structure is linked to altered postural control, reflected by larger displacements of the body’s center of mass and increased mediolateral deviations of the center of pressure during quiet standing. Rather than being a purely local foot phenomenon, such asymmetry may act as a system-level perturbation to balance regulation: when left–right plantar loading becomes uneven, the central nervous system receives asymmetric proprioceptive and plantar cutaneous input, which may increase corrective motor output and raise the neuromuscular “cost” required to maintain a stable stance^25^.

Biomechanically, arch-height asymmetry can modify the coupling between the rearfoot and midfoot, and may shift the activation strategy of key stabilizers (e.g., posterior tibial and fibular longus) to compensate for unequal medial–lateral support^26^. These compensations are plausibly amplified when asymmetry co-exists with more pronounced morphological deformation on one side, because the contralateral limb must counterbalance the altered base-of-support mechanics. Clinically, the concept that restoring bilateral alignment can improve functional control is consistent with reports that simultaneous correction of hallux valgus and flexible pes planus is accompanied by improvements in multiple radiographic alignment indices over follow-up^27^. Although our data are cross-sectional and derived from standardized static tasks, they support the interpretation that arch asymmetry is a meaningful biomechanical correlate of postural regulation, motivating future longitudinal studies to determine whether reducing asymmetry translates into measurable improvements in balance-related outcomes.

A notable feature of our dataset is that associations were consistently stronger in the left foot. One plausible hypothesis is functional laterality: if most participants are right-foot dominant, the right limb may exhibit stronger neuromuscular control and task-specific compensation, while the left limb serves more often as a stabilizing/support limb during daily activities—potentially making structural deviations more evident under quiet standing assessments. This laterality interpretation should be tested explicitly in future work by recording dominance and by including dynamic tasks that stress limb-specific roles.

### Regulation of elastic energy storage systems by arch variations

Our findings indicate that abnormal arch morphology is associated with reduced elastic energy storage and recoil capacity, as reflected by lower AEI and PRR in deformed arches. Conceptually, AEI and PRR capture complementary aspects of the arch’s “spring-like” behavior: AEI relates to the ability of the arch to store elastic deformation energy under load, whereas PRR reflects the efficiency and rate at which plantar loading recovers toward baseline during unloading. In this framework, the pronounced reduction in these indices in severe flatfoot suggests an impaired arch-spring mechanism.

Mechanistically, two pathways may contribute. First, chronic arch collapse can alter the tensioning behavior of the plantar fascia and associated ligaments, reducing effective pre-tension and changing how load is redistributed across the forefoot and midfoot^27,28^. Recent biomechanical modeling further supports the idea that the plantar fascia and long plantar ligaments play coordinated roles in supporting the medial longitudinal arch under body weight; disruption of this load-sharing system could plausibly degrade elastic energy management in flattened arches^29,30^. Second, compensatory overuse of the posterior tibial tendon and medial structures may shift the pressure trajectory anteriorly and medially, thereby reducing efficient energy return and amplifying localized forefoot loading during propulsion^5^.

High-arched feet al.so showed reduced AEI, which may reflect a different mechanical limitation: a stiffer, less deformable arch can store less energy by deformation, and impact forces may be transmitted more directly to proximal joints^2^. This interpretation is also consistent with prior evidence that cavus-type (high-arched) feet can exhibit greater forefoot plantar-pressure loading under weight-bearing conditions, supporting a plausible forefoot-overload pathway in rigid-arch phenotypes^1^. Consistent with this interpretation, finite element analysis during running demonstrates that interventions that reduce plantar fascial strain (e.g., taping) can increase navicular height and alter strain patterns, underscoring the sensitivity of the arch-spring system to small changes in structural support and loading^31^. Together, these findings support a measured interpretation that both flattened and highly arched morphologies may compromise elastic energy regulation, but through partially distinct mechanical routes (excess compliance vs. excessive stiffness).

### Impact of arch asymmetry on gait coordination and dynamic stability

Arch asymmetry was also associated with altered inter-limb coordination and sway dynamics, reflected by higher CAI and shifts in the temporal structure of COP fluctuations (Hurst exponent). Importantly, CAI and Hurst in the present study were derived from quiet standing COP time-series; therefore, these indices should be interpreted as markers of postural coordination and stability-related control, rather than direct measures of gait performance. Within this scope, greater left–right discrepancies in arch type or height may indicate that the two feet operate with different mechanical constraints, requiring asymmetric control strategies to maintain a stable base of support.

From a mechanical perspective, asymmetric arch support can bias how the COP migrates between rearfoot and forefoot regions and may disrupt the usual sequencing of load transfer across the stance footprint. This interpretation is consistent with in vitro evidence that posterior tibial tendon dysfunction alters plantar pressure patterns and shifts the COP trajectory, increasing abnormal loading on the medial forefoot^5^. From a neuromechanical perspective, a lower Hurst exponent suggests less structured, more “random-like” sway behavior, which may reflect increased corrective adjustments or reduced efficiency of sensorimotor integration. These observations align with the broader proposition that chronic foot dysfunction can contribute to compensatory changes up the kinetic chain, potentially influencing knee and lumbopelvic mechanics over time^3,4^.

Taken together, our data support the measured conclusion that arch asymmetry is associated with less coordinated and less stable postural control signatures under standardized standing conditions. Future studies should test whether these static coordination signatures predict dynamic performance by incorporating gait tasks, sport-specific maneuvers, and synchronized neuromuscular recordings, thereby clarifying how static asymmetry relates to movement-level stability.

### Sports practice and clinical rehabilitation implications​​

Importantly, the following implications are intended as screening cues derived from standardized quiet-standing assessments rather than prescriptive treatment or injury-risk guidance. Static standing correlates do not directly translate to dynamic injury risk or sport-specific performance, and any intervention-oriented implications should be considered hypothesis-generating pending prospective and interventional confirmation.

Our results support expanding arch screening beyond categorical “type” toward a combined framework of type + function + asymmetry in university populations. (1) Pre-participation screening: annual fitness assessments may prioritize individuals with more pronounced structural deformation or lower functional indices (AEI/PRR), which may reflect reduced shock attenuation or elastic recoil capacity and serve as cues for follow-up assessment and individualized discussion of training exposure and footwear habits, rather than direct guidance. (2) Low-arch phenotype: for individuals with low arches accompanied by marked AEI/PRR impairment, intrinsic foot muscle strengthening (e.g., short-foot exercises) may be considered a candidate target for future intervention testing; prior interventions have reported improvements in arch support–related outcomes^32,33^. Linking to our data, the pronounced AEI/PRR reductions observed in the most deformed flatfoot subgroup suggest a “reduced energy storage/recovery” phenotype that may be prioritized for further evaluation in interventional research. (3) High-arch phenotype: for high-arched individuals with forefoot overload patterns (e.g., hallux valgus tendency or forefoot symptoms), mobility training and footwear with enhanced cushioning may be explored as potentially supportive factors in individualized evaluation, but should not be interpreted as actionable recommendations based solely on static screening. (4) Asymmetry phenotype: when structural asymmetry co-occurs with coordination-related abnormalities (e.g., elevated CAI), orthotic strategies may be complemented by sensory and balance training to address a potentially higher postural-control burden—although prospective and interventional studies are required to confirm effects on injury- or pain-related endpoints.

### Limitations and future directions​​

This study should be interpreted in light of several limitations. First, the cross-sectional design and the absence of injury-incidence data preclude causal inference and do not allow direct estimation of injury risk; our findings therefore represent associations between arch morphology and biomechanical correlates measured under standardized conditions. Second, participants were recruited from a single university via an annual fitness/health assessment program, which may introduce selection bias and limit generalizability to students with different training exposures or clinical profiles. Because participation was voluntary within a university screening context, the sample may overrepresent students who are more health-conscious or more willing/able to complete testing, further limiting broader generalizability. Third, several potentially important confounders were not measured or controlled, including habitual physical activity and sport type, training volume, habitual footwear, lower-limb strength, and adiposity-related factors; although body weight was recorded, height/body composition were not systematically available for analytical adjustment (e.g., BMI), and residual confounding may therefore influence the observed associations. Sensitivity-oriented interpretation: because these unmeasured factors are plausibly associated with both arch morphology and static outcomes, residual confounding could bias estimates in either direction (inflating or attenuating associations). In this screening cohort, the likely impact would be small-to-moderate shifts in effect estimates for many outcomes, but larger bias cannot be excluded for measures that are highly training- or adiposity-sensitive. In addition, footedness (limb dominance) was not recorded, limiting our ability to directly test dominance-related explanations for the observed laterality patterns, and therefore laterality explanations should be interpreted cautiously.

Methodologically, all biomechanical assessments were performed during quiet standing, which provides a pragmatic screening snapshot but may not capture gait- or sport-specific dynamics. Accordingly, static standing correlates should not be interpreted as direct indicators of dynamic injury risk. Moreover, although scanning posture was standardized, subtle left–right differences in weight distribution or platform interaction could contribute to side-related measurement bias; future work should explicitly quantify potential systematic scanner-side effects using repeated scans with controlled loading (and/or cross-validation against established indices). Because the primary analytical dataset was curated at the level of averaged trial outputs, within-session reliability indices (e.g., ICC and SEM) could not be quantified from trial-level data in the present report; averaging was used pragmatically to reduce within-session noise under standardized posture, and future studies should retain and report trial-resolved outputs to enable formal reliability estimation (ICC/SEM) alongside averaged measures. In addition, estimates for less prevalent arch categories (e.g., high-arched feet) may be more sensitive to influential observations and should be interpreted with caution until replicated in independent cohorts and/or confirmed via prespecified influence diagnostics. This caution may be particularly relevant for the hallux valgus estimate in the high-arch subgroup. Future studies should incorporate motion capture–based gait analysis, in-shoe pressure measurement, and synchronized force-plate kinetics and surface electromyography to test whether the static coupling patterns observed here persist during mid-stance and propulsion, and to clarify neuromuscular control strategies during locomotion and sport tasks^34,35^.

To make the next steps more actionable, two study designs may be particularly informative: (i) a prospective follow-up within annual fitness programs that tracks injury incidence and pain outcomes, with a priori stratification of students showing marked bilateral asymmetry (e.g., arch height difference > 3.2 mm and/or elevated coordination asymmetry), and (ii) randomized controlled trials testing whether customized orthoses and/or targeted intrinsic-foot and balance training improves AEI/PRR and coordination-related outcomes (CAI/Hurst) over time, alongside clinically meaningful endpoints (symptoms, function, or injury surveillance). Finally, longitudinal follow-up of students—particularly those with marked bilateral arch asymmetry—together with prespecified confirmatory analyses in independent cohorts, would help clarify temporal relationships and test whether modifying arch-related mechanics leads to meaningful functional improvements.

## Conclusion

In this large cross-sectional cohort of university students, foot arch morphology was associated with multiple static biomechanical domains, including forefoot and rearfoot alignment characteristics, arch-related functional indices (AEI and PRR), and postural-control surrogates that reflected bilateral asymmetry. Importantly, both low-arched and high-arched phenotypes showed less favorable profiles in different domains, supporting a non-linear perspective that extends screening beyond a flatfoot-only focus. In addition, greater bilateral arch asymmetry was consistently linked to altered static stability signatures, suggesting that inter-limb structural differences may be an informative marker of increased postural-control demands under standardized standing conditions.

Collectively, these findings support a practical screening framework that integrates arch type, arch function, and asymmetry within university fitness assessments, with potential relevance for load-management counseling, footwear/orthotic decision-making, and targeted exercise or balance-oriented rehabilitation strategies. However, because measurements were obtained during quiet standing and the study design was cross-sectional, the observed relationships should be interpreted as associations rather than causal effects. Prospective follow-up and randomized interventions are warranted to determine whether modifying arch-related mechanics (e.g., via customized orthoses or structured exercise programs) leads to improvements in AEI/PRR, coordination-related indices, symptoms, and injury-relevant outcomes across broader populations and task contexts. Finally, we emphasize that these quiet-standing correlates should not be interpreted as direct indicators of dynamic injury risk.

## Supplementary Information

Below is the link to the electronic supplementary material.

Supplementary Material 1

## Data Availability

Data is provided within the manuscript or supplementary information files.
