# Supplementary material for: Foot arch morphology and lower-limb biomechanical characteristics in university students: a cross-sectional multifactorial analysis of 1,078 participants
Source: Sci Rep. 2026 Feb 5;16:7329. doi: 10.1038/s41598-026-38118-1 (PMC12923694; doi:10.1038/s41598-026-38118-1)
Supplement: Supplementary file 1 — Supplementary Material 1 [file 41598_2026_38118_MOESM1_ESM.docx]

**SUPPLEMENT**

**Supplementary Table 1.** Multivariate Regression Analysis of Arch Morphology Effects on Bilateral Hallux Valgus and Calcaneal Inclination Angles

| Model | Variables / Statistics | *B* (Unstandardized Coefficient) | SE (Standard Error) | *Beta* (Standardized Coefficient) | *t*-value | *P*-value | 95% CI (Confidence Interval) | *VIF* | *R*² | Adjusted *R*² | Standard Error | *F*-value (df) |
| --- | --- | --- | --- | --- | --- | --- | --- | --- | --- | --- | --- | --- |
| Hallux Valgus Angle (Left) Regression Results | | | | | | | | | | | | |
| Continuous Model | Arch height (Left) | −1.105 | 0.111 | −0.289 | −9.91 | 0.001 | [−1.324, −0.886] | 1 | - | - | - | - |
|  | Model Statistics | - | - | - | - | <0.001 | - | - | 0.084 | 0.083 | 9.897 | 98.209 (1,1076) |
| Categorical Model | Left Flatfoot | 14.928 | 0.432 | 0.589 | 34.546 | <0.001 | [14.080, 15.776] | 1.31 | - | - | - | - |
|  | Left Flatfoot+ | 15.664 | 0.432 | 0.619 | 36.295 | <0.001 | [14.817, 16.510] | 1.311 | - | - | - | - |
|  | Left Flatfoot++ | 18.08 | 0.428 | 0.722 | 42.223 | <0.001 | [17.240, 18.921] | 1.316 | - | - | - | - |
|  | Left High-Arched Foot | 35.303 | 0.801 | 0.684 | 44.069 | <0.001 | [33.731, 36.875] | 1.084 | - | - | - | - |
|  | Model Statistics | - | - | - | - | <0.001 | - | - | 0.762 | 0.761 | 5.053 | 857.651 (4,1073) |
| Hallux Valgus Angle (Right) Regression Results | | | | | | | | | | | | |
| Continuous Model | Arch height (Right) | -0.483 | 0.097 | −0.151 | -4.998 | 0.001 | [-0.672, -0.293] | 1 | - | - | - | - |
|  | Model Statistics | - | - | - | - | <0.001 | - | - | 0.023 | 0.022 | 8.41 | 24.982 (1,1076) |
| Categorical Model | Right Flatfoot | 4.47 | 0.642 | 0.221 | 6.96 | <0.001 | [3.210, 5.731] | 1.277 | - | - | - | - |
|  | Right Flatfoot+ | 5.366 | 0.694 | 0.242 | 7.728 | <0.001 | [4.004, 6.729] | 1.242 | - | - | - | - |
|  | Right Flatfoot++ | 7.678 | 0.675 | 0.358 | 11.382 | <0.001 | [6.354, 9.001] | 1.256 | - | - | - | - |
|  | Right High-Arched Foot | 11.129 | 1.17 | 0.278 | 9.511 | <0.001 | [8.833, 13.426] | 1.082 | - | - | - | - |
|  | Model Statistics | - | - | - | - | <0.001 | - | - | 0.153 | 0.149 | 7.842 | 48.284 (4,1073) |
| Calcaneal Inclination Angle (Left) Regression Results | | | | | | | | | | | | |
| Continuous Model | Arch height (Left) | -2.447 | 0.034 | -0.91 | -72.072 | <0.001 | [-2.513, -2.380] | 1 | - | - | - | - |
|  | Model Statistics | - | - | - | - | <0.001 | - | - | 0.828 | 0.828 | 3.013 | 5194.409 (1,1076) |
| Categorical Model | Left Flatfoot | 5.582 | 0.288 | 0.313 | 19.402 | <0.001 | [5.017, 6.146] | 1.31 | - | - | - | - |
|  | Left Flatfoot+ | 8.943 | 0.287 | 0.502 | 31.125 | <0.001 | [8.379, 9.506] | 1.311 | - | - | - | - |
|  | Left Flatfoot++ | 15.412 | 0.285 | 0.874 | 54.06 | <0.001 | [14.852, 15.971] | 1.316 | - | - | - | - |
|  | Left High-Arched Foot | -8.659 | 0.533 | -0.238 | −16.236 | <0.001 | [−9.706, −7.613] | 1.084 | - | - | - | - |
|  | Model Statistics | - | - | - | - | <0.001 | - | - | 0.787 | 0.786 | 3.364 | 989.356 (4,1073) |
| Calcaneal Inclination Angle (Right) Regression Results | | | | | | | | | | | | |
| Continuous Model | Arch height (Right) | -2.252 | 0.032 | −0.908 | −71.026 | <0.001 | [−2.314, −2.190] | 1 | - | - | - | - |
|  | Model Statistics | - | - | - | - | <0.001 | - | - | 0.824 | 0.824 | 2.762 | 5044.681 (1,1076) |
| Categorical Model | Right Flatfoot | 3.537 | 0.252 | 0.226 | 14.05 | <0.001 | [3.043, 4.031] | 1.277 | - | - | - | - |
|  | Right Flatfoot+ | 8.979 | 0.272 | 0.523 | 32.989 | <0.001 | [8.445, 9.513] | 1.242 | - | - | - | - |
|  | Right Flatfoot++ | 13.272 | 0.264 | 0.8 | 50.2 | <0.001 | [12.753, 13.791] | 1.256 | - | - | - | - |
|  | Right High-Arched Foot | -8.499 | 0.459 | -0.274 | -18.529 | <0.001 | [−9.398, −7.599] | 1.082 | - | - | - | - |
|  | Model Statistics | - | - | - | - | <0.001 | - | - | 0.783 | 0.782 | 3.074 | 967.308 (4,1073) |

**Supplementary Table 2.** Bilateral Arch Asymmetry Effects on Postural Stability: Multivariate Regression Analysis

| Variables / Statistics | *B* (Unstandardized Coefficient) | SE (Standard Error) | *Beta* (Standardized Coefficient) | *t*-value | *P*-value | 95% CI (Confidence Interval) | *VIF* | *R*² | Adjusted *R*² | Standard Error | *F*-value (df) |
| --- | --- | --- | --- | --- | --- | --- | --- | --- | --- | --- | --- |
| Arch Disparity → Center-of-Mass Displacement | | | | | | | | | | | |
| Bilateral Arch Height Difference | 10.725 | 0.335 | 0.698 | 31.976 | <0.001 | [10.067, 11.383] | 1 | - | - | - | - |
| Model Statistics | - | - | - | - | <0.001 | - | - | 0.487 | 0.487 | 10.996 | 1022.475(1,1076) |
| Arch Disparity → Mediolateral Pressure Center Deviation | | | | | | | | | | | |
| Bilateral Arch Height Difference | 1.58 | 0.053 | 0.672 | 29.741 | <0.001 | [1.476, 1.684] | 1 | - | - | - | - |
| Model Statistics | - | - | - | - | <0.001 | - | - | 0.451 | 0.451 | 2.63 | 884.512(1,1076) |

**Supplementary Table 3.** Arch Morphology Effects on AEI and PRR: Multivariate Regression Analysis (Bilateral)

| Variables / Statistics | *B* (Unstandardized Coefficient) | SE (Standard Error) | *Beta* (Standardized Coefficient) | *t*-value | *P*-value | 95% CI (Confidence Interval) | *VIF* | *R*² | Adjusted *R*² | Standard Error | *F*-value (df) |
| --- | --- | --- | --- | --- | --- | --- | --- | --- | --- | --- | --- |
| Arch Elasticity Index (AEI) – Left Foot | | | | | | | | | | | |
| Left Flatfoot | −18.373 | 0.518 | −0.303 | −35.435 | <0.001 | [−19.390, −17.355] | 1.31 | - | - | - | - |
| Left Flatfoot+ | −42.002 | 0.518 | −0.693 | −81.116 | <0.001 | [−43.018, −40.986] | 1.311 | - | - | - | - |
| Left Flatfoot++ | −62.371 | 0.514 | −1.039 | −121.393 | <0.001 | [−63.379, −61.363] | 1.316 | - | - | - | - |
| Left High-Arched Foot | −41.859 | 0.961 | −0.338 | −43.55 | <0.001 | [−43.745, −39.973] | 1.084 | - | - | - | - |
| Model Statistics | - | - | - | - | <0.001 | - | - | 0.94 | 0.94 | 6.063 | 4219.588(4,1073) |
| Arch Elasticity Index (AEI) – Right Foot | | | | | | | | | | | |
| Right Flatfoot | −19.785 | 0.453 | −0.343 | −43.641 | <0.001 | [−20.675, −18.896] | 1.277 | - | - | - | - |
| Right Flatfoot+ | −43.311 | 0.49 | −0.685 | −88.362 | <0.001 | [−44.272, −42.349] | 1.242 | - | - | - | - |
| Right Flatfoot++ | −61.891 | 0.476 | −1.014 | −129.986 | <0.001 | [−62.825, −60.956] | 1.256 | - | - | - | - |
| Right High-Arched Foot | −37.356 | 0.826 | −0.327 | −45.225 | <0.001 | [−38.977, −35.735] | 1.082 | - | - | - | - |
| Model Statistics | - | - | - | - | <0.001 | - | - | 0.948 | 0.948 | 5.535 | 4894.670(4,1073) |
| Pressure Recovery Rate (PRR) – Left Foot | | | | | | | | | | | |
| Left Flatfoot | −0.143 | 0.003 | −0.302 | −49.902 | <0.001 | [−0.149, −0.137] | 1.31 | - | - | - | - |
| Left Flatfoot+ | −0.27 | 0.003 | −0.572 | −94.406 | <0.001 | [−0.276, −0.264] | 1.311 | - | - | - | - |
| Left Flatfoot++ | −0.513 | 0.003 | −1.097 | −180.693 | <0.001 | [−0.518, −0.507] | 1.316 | - | - | - | - |
| Left High-Arched Foot | −0.301 | 0.005 | −0.313 | −56.781 | <0.001 | [−0.312, −0.291] | 1.084 | - | - | - | - |
| Model Statistics | - | - | - | - | <0.001 | - | - | 0.97 | 0.97 | 0.033 | 8657.550(4,1073) |
| Pressure Recovery Rate (PRR) – Right Foot | | | | | | | | | | | |
| Right Flatfoot | −0.135 | 0.004 | −0.29 | −37.074 | <0.001 | [−0.142, −0.127] | 1.277 | - | - | - | - |
| Right Flatfoot+ | −0.308 | 0.004 | −0.606 | −78.576 | <0.001 | [−0.316, −0.301] | 1.242 | - | - | - | - |
| Right Flatfoot++ | −0.508 | 0.004 | −1.032 | −133.098 | <0.001 | [−0.515, −0.500] | 1.256 | - | - | - | - |
| Right High-Arched Foot | −0.312 | 0.007 | −0.339 | −47.182 | <0.001 | [−0.325, −0.299] | 1.082 | - | - | - | - |
| Model Statistics | - | - | - | - | <0.001 | - | - | 0.949 | 0.948 | 0.044 | 4956.489(4,1073) |

**Supplementary Table 4.** Arch Morphology Effects on CAI and Hurst Index: Multivariate Regression Analysis

| Variables / Statistics | *B* (Unstandardized Coefficient) | SE (Standard Error) | *Beta* (Standardized Coefficient) | *t*-value | *P*-value | 95% CI (Confidence Interval) | *VIF* | *R*² | Adjusted *R*² | Standard Error | *F*-value (df) |
| --- | --- | --- | --- | --- | --- | --- | --- | --- | --- | --- | --- |
| Coordination Asymmetry Index (CAI) | | | | | | | | | | | |
| Left Flatfoot | 0.141 | 0.019 | 0.168 | 7.433 | <0.001 | [0.104, 0.178] | 1.626 |  |  |  |  |
| Left Flatfoot+ | 0.118 | 0.022 | 0.141 | 5.446 | <0.001 | [0.076, 0.161] | 2.14 |  |  |  |  |
| Left Flatfoot++ | 0.302 | 0.026 | 0.364 | 11.621 | <0.001 | [0.251, 0.353] | 3.128 |  |  |  |  |
| Left High-Arched Foot | 0.288 | 0.034 | 0.168 | 8.388 | <0.001 | [0.221, 0.355] | 1.285 |  |  |  |  |
| Right Flatfoot | 0.051 | 0.018 | 0.062 | 2.761 | 0.006 | [0.015, 0.087] | 1.626 |  |  |  |  |
| Right Flatfoot+ | 0.147 | 0.022 | 0.165 | 6.668 | <0.001 | [0.104, 0.191] | 1.96 |  |  |  |  |
| Right Flatfoot++ | 0.162 | 0.027 | 0.189 | 6.113 | <0.001 | [0.110, 0.215] | 3.032 |  |  |  |  |
| Right High-Arched Foot | 0.341 | 0.033 | 0.212 | 10.442 | <0.001 | [0.277, 0.406] | 1.313 |  |  |  |  |
| Bilateral Arch Type Disparity | 0.41 | 0.016 | 0.596 | 26.253 | <0.001 | [0.380, 0.441] | 1.642 |  |  |  |  |
| Bilateral Arch Height Difference | 0.026 | 0.008 | 0.077 | 3.472 | <0.001 | [0.011, 0.041] | 1.55 |  |  |  |  |
| Model Statistics | - | - | - | - | <0.001 | - | - | 0.665 | 0.662 | 0.199 | 212.119 (10,1067) |
| Hurst Index | | | | | | | | | | | |
| Left Flatfoot | −0.111 | 0.008 | −0.243 | −13.267 | <0.001 | [−0.128, −0.095] | 1.626 |  |  |  |  |
| Left Flatfoot+ | −0.156 | 0.01 | −0.339 | −16.168 | <0.001 | [−0.174, −0.137] | 2.14 |  |  |  |  |
| Left Flatfoot++ | −0.21 | 0.012 | −0.463 | −18.261 | <0.001 | [−0.233, −0.188] | 3.128 |  |  |  |  |
| Left High-Arched Foot | 0.135 | 0.015 | 0.144 | 8.88 | <0.001 | [0.105, 0.165] | 1.285 |  |  |  |  |
| Right Flatfoot | -0.107 | 0.008 | -0.241 | -13.153 | <0.001 | [-0.123, -0.091] | 1.626 |  |  |  |  |
| Right Flatfoot+ | -0.124 | 0.01 | -0.255 | -12.675 | <0.001 | [-0.144, -0.105] | 1.96 |  |  |  |  |
| Right Flatfoot++ | -0.201 | 0.012 | -0.425 | -17.017 | <0.001 | [-0.224, -0.177] | 3.032 |  |  |  |  |
| Right High-Arched Foot | 0.133 | 0.015 | 0.151 | 9.17 | <0.001 | [0.105, 0.161] | 1.313 |  |  |  |  |
| Bilateral Arch Type Disparity | 0.019 | 0.007 | 0.05 | 2.715 | 0.007 | [0.005, 0.032] | 1.642 |  |  |  |  |
| Bilateral Arch Height Difference | -0.023 | 0.003 | -0.125 | -7.015 | <0.001 | [-0.030, -0.017] | 1.55 |  |  |  |  |
| Model Statistics | - | - | - | - | <0.001 | - | - | 0.78 | 0.778 | 0.088 | 379.184 (10,1067) |
